# Genomic Stress and DNA Repair During Macrophage Differentiation and Inflammatory Activation

**DOI:** 10.3390/ijms27188276

**Published:** 2026-09-17

**Authors:** Seo-Gyeong Jo, Jeseok Jeon, Yeon-Ji Jeon, Sun-Ju Park, Tae-Hong Kang

**Affiliations:** Department of Biomedical Sciences, Dong-A University, Busan 49315, Republic of Korea; skcho9822@donga.ac.kr (S.-G.J.); 2577615@donga.ac.kr (J.J.); 2304792@donga.ac.kr (Y.-J.J.); 2350259@donga.ac.kr (S.-J.P.)

**Keywords:** macrophages, genome maintenance, DNA repair, DNA damage response, nucleotide excision repair, immunometabolism

## Abstract

Macrophages must preserve genome stability while performing immune and tissue-supporting functions that can themselves induce DNA damage or interfere with genome maintenance. Inflammatory metabolism produces reactive oxygen and nitrogen species, extensive transcription imposes topological and transcription-associated stress, and proliferative responses generate replication stress. At the same time, studies of monocyte-derived macrophages and macrophage-like differentiation models show selective changes in DNA repair capacity: base excision repair (BER)/single-strand break (SSB) repair and DNA-dependent protein kinase (DNA-PK)-dependent double-strand break (DSB) repair are enhanced, whereas global nucleotide excision repair (NER) can be attenuated while repair is preferentially retained in transcriptionally active regions. When these protective mechanisms fail, persistent DNA damage can promote apoptosis or senescence, alter inflammatory signaling and extracellular communication, and modify self-antigen presentation. Here, we focus on differentiation-associated changes in DNA repair capacity and genome maintenance during inflammatory activation, drawing on selected tissue and disease models. We also discuss emerging evidence that circadian regulation of metabolism and redox activity may add a temporal dimension to this balance. Defining how pathway-specific DNA repair capacity is matched to state-dependent genotoxic challenges will clarify how genome stability supports immune homeostasis and how its disruption contributes to inflammatory dysfunction.

## 1. Introduction

Macrophages inhabit diverse tissue environments and continuously adapt to local signals through coordinated changes in transcription, metabolism, phagocytosis, cytokine production, and interactions with neighboring cells [1,2,3]. This functional plasticity supports transitions between surveillance, antimicrobial defense, clearance of damaged cells, and tissue repair [4], accompanied by substantial changes in cellular metabolism and transcription [5]. Comparative transcriptional and epigenomic studies have established that tissue-resident macrophages acquire distinct sensing, metabolic, and functional programs in response to local cytokines, metabolites, stromal interactions, and environmental exposure [6,7]. These state-dependent changes in transcription and metabolism also influence the spectrum and burden of DNA damage encountered by macrophages.

**Figure 1:**
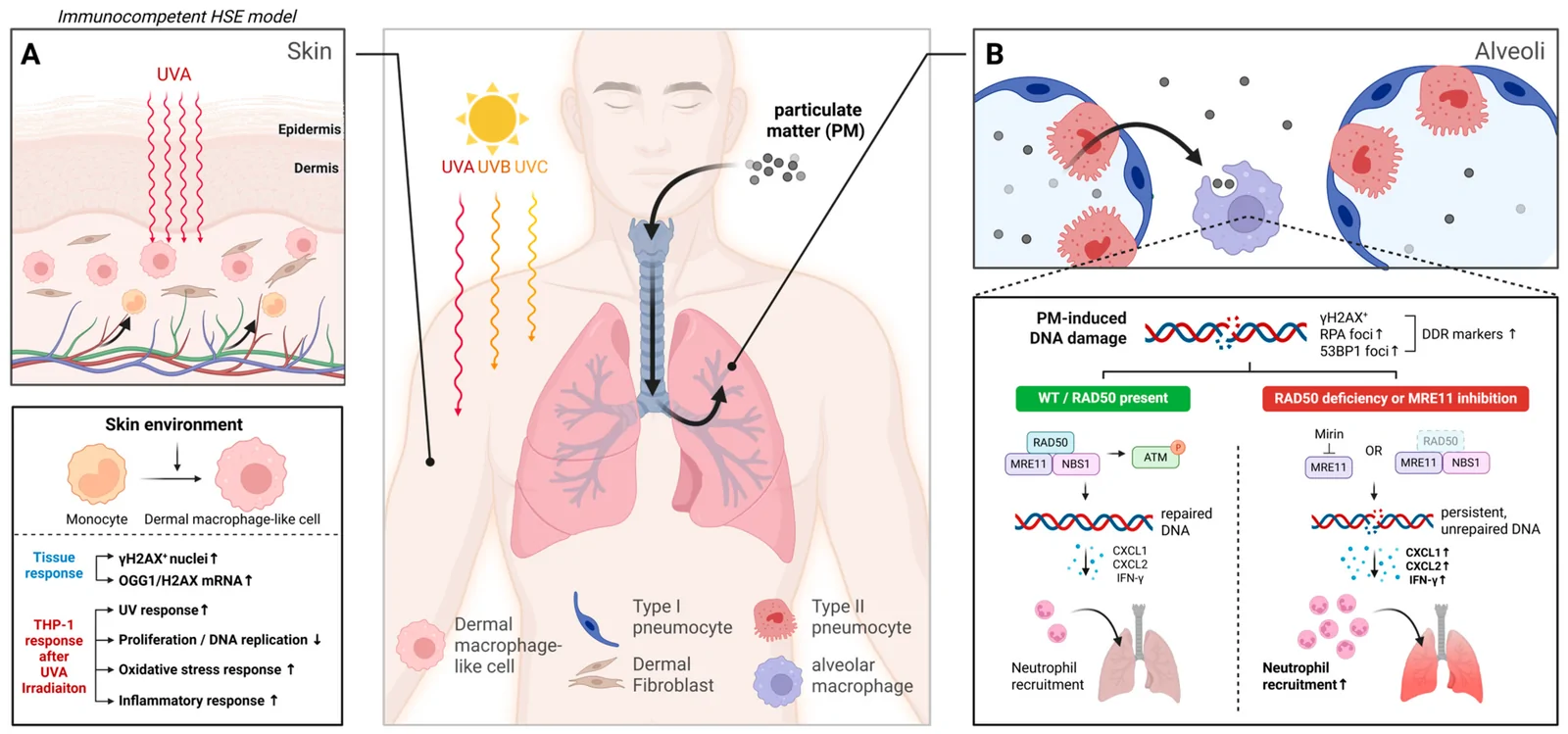
**Tissue-specific DNA damage responses in macrophages.** (**A**) THP-1 cells acquire macrophage-like features in a three-dimensional human skin-equivalent (HSE) model and exhibit DNA damage, oxidative stress, and inflammatory responses after ultraviolet A (UVA) exposure. (**B**) Particulate matter (PM) activates the MRE11-RAD50-NBS1 (MRN) DNA damage response in alveolar macrophages; impaired MRN function increases unrepaired DNA and airway inflammation. The panels depict distinct experimental settings, not a shared precursor-to-tissue-macrophage differentiation pathway. Created in BioRender. Jo, S. (2026) https://BioRender.com/esiznoq.

In a three-dimensional human skin-equivalent (HSE) model, THP-1 monocytic cells incorporated into the dermal compartment acquired macrophage-like phenotypic and transcriptional features distinct from those of conventional two-dimensional THP-1 cultures, including enrichment of extracellular matrix interaction programs and reduced DNA replication programs. Following ultraviolet A (UVA) exposure, these HSE-associated macrophage-like cells showed increased DNA damage markers together with oxidative stress and inflammatory responses [8]. In the lung, particulate matter (PM) exposure induces DNA damage in alveolar macrophages and activates an MRE11–RAD50–Nijmegen breakage syndrome protein 1 (NBS1) complex (MRN)-dependent DNA damage response, accompanied by replication protein A (RPA), p53-binding protein 1 (53BP1), and phosphorylated histone H2AX (γH2AX) foci and ataxia-telangiectasia mutated (ATM) activation. This response contributes to the control of PM-induced DNA damage and limits the associated inflammatory response; consistent with this, RAD50 deficiency or MRE11 inhibition results in persistent unrepaired DNA, enhanced inflammatory cytokine production, and increased airway neutrophil recruitment [9] (Figure 1). Together, these models illustrate how tissue context and environmental exposure jointly shape macrophage phenotype and DNA damage responses. Whether tissue-resident macrophage populations also differ intrinsically in DNA repair capacity remains largely unknown.

Many tissue-resident macrophage populations are long-lived and capable of local self-maintenance [10]. Genome integrity must therefore be preserved during repeated or prolonged episodes of macrophage activity rather than during a single transient response. At the same time, several processes required for macrophage function can themselves induce DNA damage or otherwise threaten genome integrity. Macrophage genome integrity can therefore be viewed as a balance between state-dependent insults to genome integrity and the DNA damage signaling and repair capacity available to counter them.

Developmental origin and tissue identity must be distinguished from monocyte-to-macrophage differentiation when interpreting DNA repair phenotypes. Kupffer cells in the liver, microglia in the central nervous system, and distinct splenic and connective-tissue macrophage populations occupy distinct niches. These populations cannot be represented by a single differentiation model, and many resident macrophages self-maintain with limited input from circulating monocytes [4,6,7,10]. Accordingly, Figure 1 illustrates selected exposure models rather than a general developmental sequence. This Review focuses on differentiation-dependent changes in DNA repair capacity and on inflammatory activation, rather than providing an exhaustive comparison of DNA repair across anatomical macrophage populations.

In addition to developmental origin and tissue identity, activation state represents an independent source of macrophage heterogeneity. The conventional M1 and M2 labels describe broad inflammatory and alternatively activated phenotypes, but do not encompass the mixed and changing states found in tissues [3,5]. We therefore specify stimuli, including interferon gamma (IFN-γ)/lipopolysaccharide (LPS) and interleukin-4 (IL-4), where available, rather than assigning a fixed DNA repair phenotype to either category. Differentiation-dependent changes in DNA repair capacity and activation-dependent changes in DNA damage burden should consequently be evaluated separately.

Several macrophage activities expose the genome to distinct forms of DNA damage, replication stress, and transcription-associated stress. Inflammatory and antimicrobial activation generates reactive oxygen species (ROS) and reactive nitrogen species (RNS), including mitochondrial ROS and nitric oxide, which can produce oxidative and deamination-associated DNA lesions [11,12,13]. Extensive transcription imposes a different challenge. Rapid induction of inflammatory and tissue-response genes requires chromatin remodeling, sustained RNA polymerase II progression, and control of DNA topology [14]. DNA topoisomerase I (TOP1), for example, supports transcription of pathogen-induced genes in macrophages [15]. Replication stress arises more selectively when macrophages enter the cell cycle during local expansion, inflammation, or tissue repair. Thus, redox and metabolic activity, transcription, and DNA replication represent distinct threats to genome integrity whose relative contributions vary according to macrophage state.

Unlike adaptive lymphocytes, which deliberately generate DNA lesions during antigen-receptor diversification, macrophages do not normally rely on programmed genome rearrangement [16]. Instead, DNA lesions, replication stress, and transcription-associated stress arise largely as consequences of macrophage metabolism, inflammatory signaling, transcription, and proliferation. DNA damage responses and repair must therefore preserve genome integrity without preventing the cellular activities that generate these stresses.

The DNA repair component of this balance is also selectively remodeled according to macrophage state. During monocyte-to-macrophage differentiation, base excision repair (BER), single-strand break (SSB) repair, and double-strand break repair mediated by DNA-dependent protein kinase (DNA-PK) become more efficient [17,18]. In contrast, macrophage-like differentiation is associated with attenuation of global nucleotide excision repair (NER), while repair remains comparatively efficient within transcriptionally active genomic regions [19]. Macrophage differentiation therefore does not uniformly increase or suppress DNA repair capacity; rather, individual repair pathways and genomic regions are regulated differently. Much of this mechanistic evidence, however, derives from circulating monocytes, monocyte-derived macrophages, or macrophage-like differentiation systems, and whether comparable remodeling occurs across macrophages maintained in distinct tissue environments remains insufficiently defined.

The consequences of DNA damage depend in part on whether lesions are efficiently repaired or tolerated. Defective repair during inflammatory activation can increase macrophage apoptosis and reduce persistence, whereas dysregulated DNA repair and DNA damage response signaling can contribute to macrophage senescence [20,21]. Persistent or mislocalized damaged DNA can also influence extracellular-vesicle communication and cytoplasmic DNA-sensing pathways and alter the processing and presentation of self-derived antigens [22,23]. Disease-associated macrophage states provide direct examples of these relationships. In granuloma-associated macrophages, sustained inflammatory signaling couples cell-cycle entry to replication stress, while ataxia-telangiectasia and Rad3-related (ATR)-dependent DNA damage signaling limits chromosome-segregation defects [24]. In atherosclerotic macrophages, 8-oxoguanine DNA glycosylase (OGG1)-dependent repair limits the accumulation and inflammatory consequences of oxidized mitochondrial DNA [25]. These examples show that distinct macrophage states rely on different DNA damage responses or repair pathways according to the predominant DNA lesions or replication stress in each biological context.

In addition to varying across tissue and activation states, this balance may also change over time. Circadian clock components regulate macrophage metabolism, mitochondrial ROS production, antioxidant responses, and inflammatory function [26,27]. DNA repair capacity exhibits circadian variation in several non-macrophage systems, raising the possibility that the balance between DNA damage formation and repair may also vary with time in macrophages. Direct evidence for rhythmic regulation of DNA repair capacity in macrophages, however, remains limited.

Here, we examine macrophage genome maintenance as a dynamic balance between state-dependent threats to genome integrity and the DNA damage responses and repair pathways available to counter them. We first define major sources of DNA damage, replication stress, and transcription-associated stress during macrophage activity and then examine the selective remodeling of DNA repair capacity during differentiation. We next discuss disease-associated states in which specific DNA lesions or replication stress are coupled to distinct DNA damage signaling or repair responses, followed by the inflammatory and immunological consequences of persistent or mislocalized damaged DNA. Finally, we consider tissue environment and circadian timing as potential determinants of this balance and outline the experimental approaches required to distinguish damage formation, repair, and persistent DNA damage signaling in macrophages.

## 2. Major Sources of Genomic Stress During Macrophage Activation

Macrophage activity exposes the genome to several forms of stress arising from the cellular processes required for immune and tissue functions. Redox and metabolic activity can generate oxidative and deamination-associated DNA lesions, extensive transcription requires continuous RNA polymerase II progression and control of DNA topology, and DNA replication introduces an additional source of stress when macrophages enter the cell cycle. These processes are not engaged to the same extent in every macrophage state, and their relative contributions therefore vary with inflammatory activity, metabolic state, transcriptional output, and proliferative status.

### 2.1. Redox and Metabolic Stress

Redox and metabolic activities provide a direct link between macrophage activation and DNA damage. Reactive oxygen and nitrogen species generated during inflammatory and antimicrobial responses can modify DNA bases and generate strand-break-associated intermediates [12,28]. Toll-like receptor (TLR) 1/2/4 signaling promotes mitochondrial reactive oxygen species (mtROS) production through the TRAF6-ECSIT pathway [11], while enhanced succinate oxidation through succinate dehydrogenase provides an additional source of mtROS during inflammatory activation [13]. Consistent with these mechanisms, interferon gamma (IFN-γ)/lipopolysaccharide (LPS) stimulation increases oxidative DNA lesions and γH2AX in macrophages, with mitochondrial complex III contributing substantially to mtROS production under these conditions [29]. DNA damage generated under these conditions can, in turn, affect macrophage metabolism. Poly(ADP-ribose) polymerase (PARP) activation consumes oxidized nicotinamide adenine dinucleotide (NAD^+^) during the response to DNA strand breaks, whereas activated macrophages increase nicotinamide phosphoribosyltransferase (NAMPT)-dependent NAD^+^ salvage to maintain the NAD^+^ required for GAPDH activity and inflammatory glycolysis [29]. Thus, redox and metabolic activity can both induce oxidative DNA damage and influence the metabolic capacity required to sustain macrophage inflammatory function.

### 2.2. Transcription-Associated and Topological Stress

Extensive transcription creates a different challenge for genome maintenance. Macrophage activation rapidly induces genes involved in cytokine production, antimicrobial defense, metabolism, and tissue responses [30]. Sustaining these transcriptional programs requires continued RNA polymerase II elongation, chromatin remodeling, and appropriate control of DNA topology [31]. RNA polymerase II elongation generates transcription-associated supercoiling, and TOP1 relieves the resulting torsional stress. TOP1 resolves these topological changes, and inhibition of TOP1 suppresses subsets of pathogen-induced genes in macrophages [15,32]. TLR stimulation further redistributes TOP1 to newly activated super-enhancers, where it cooperates with BRG1 to maintain chromatin accessibility and support inflammatory gene expression [33]. These findings support an important role for sustained transcriptional elongation and DNA topology control during macrophage activation, although the extent to which normal macrophage activation generates persistent transcription-associated DNA damage remains unclear.

### 2.3. Replication Stress

Replication stress becomes relevant primarily when macrophages enter the cell cycle during inflammation, tissue repair, or local expansion [10,34]. Granuloma-associated macrophages provide a direct example: persistent toll-like receptor 2 (TLR2) signaling promotes Myc-dependent cell-cycle progression while slowing replication-fork progression and increasing phosphorylated RPA, γH2AX, and 53BP1 [24]. These observations demonstrate that prolonged inflammatory signaling can couple macrophage proliferation to replication stress. The ATR-dependent response to this stress and its role during polyploid macrophage differentiation are discussed further in Section 4.1.

Together, redox and metabolic activity, extensive transcription, and DNA replication impose distinct, state-dependent challenges to genome maintenance in macrophages. These challenges are superimposed on differentiation-dependent changes in DNA repair capacity. The next section examines how macrophage differentiation selectively alters individual repair pathways.

## 3. Selective Remodeling of DNA Repair During Macrophage Differentiation

Macrophage differentiation alters DNA repair capacity in a pathway-specific rather than uniform manner. In circulating monocytes, completion of base excision repair (BER) and single-strand break (SSB) repair is inefficient, and DNA-dependent protein kinase (DNA-PK)-dependent double-strand break (DSB) repair is also limited. These activities are substantially restored during differentiation into macrophages [17,18]. Nucleotide excision repair (NER), however, follows a different pattern: global excision repair can be attenuated during macrophage differentiation, while repair remains comparatively efficient within transcriptionally active genomic regions [19,35]. Macrophage differentiation can therefore alter individual DNA repair pathways in different directions and redistribute repair capacity across the genome (Table 1).

### 3.1. Restoration of BER/SSB Repair Completion and DNA-PK-Dependent DSB Repair

**Figure 2:**
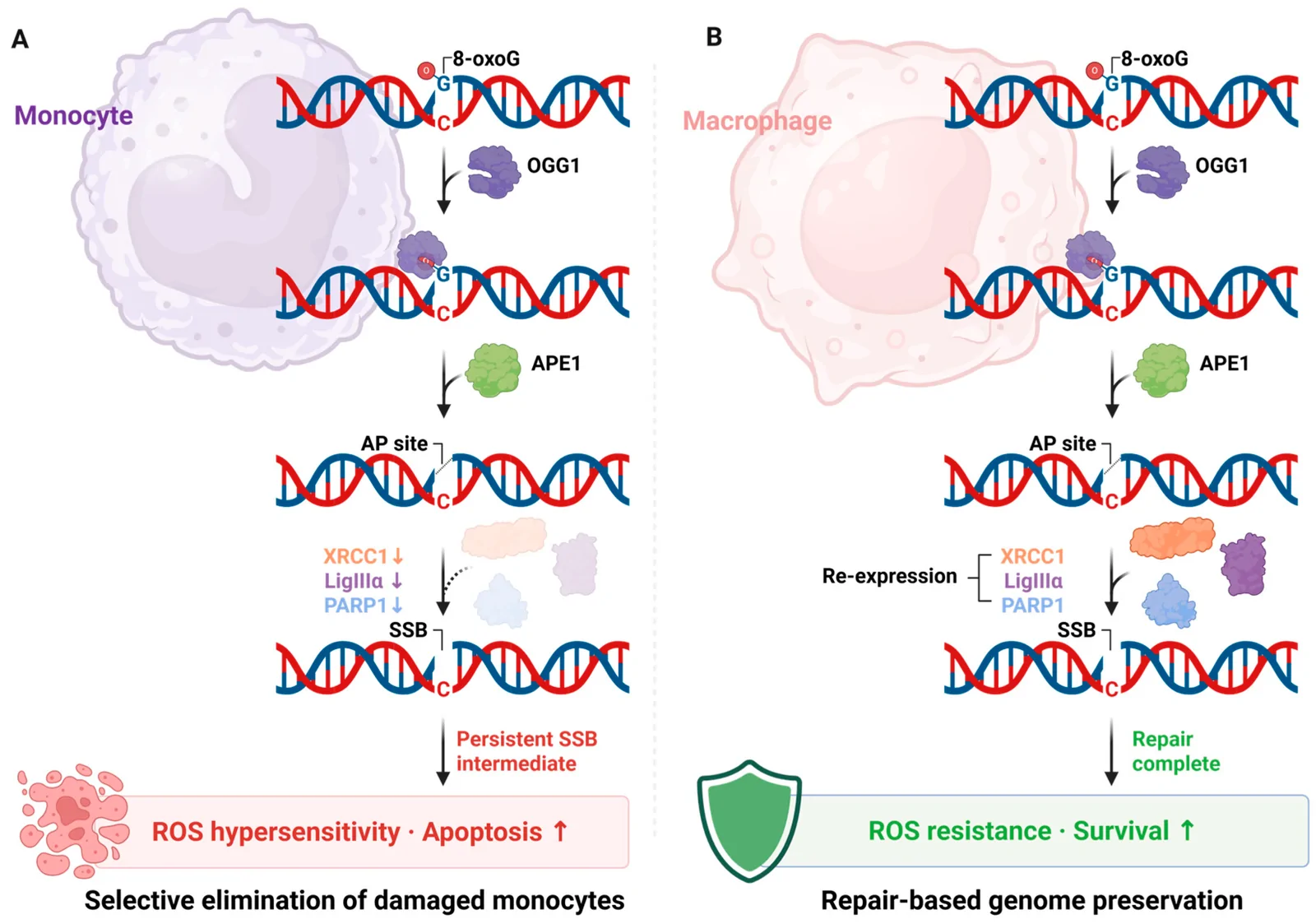
**Differentiation-associated restoration of BER/SSB repair completion in monocytes and macrophages.** (**A**) Circulating monocytes retain enzymes involved in the early steps of base excision repair (BER), including OGG1 and APE1, allowing recognition and initial processing of oxidative DNA lesions. However, low expression of downstream repair factors, including XRCC1, DNA ligase IIIα, and PARP1, limits efficient completion of BER and single-strand break (SSB) repair and can result in persistent repair intermediates. This repair-deficient state is associated with increased sensitivity to oxidative stress and enhanced apoptosis. (**B**) During differentiation into macrophages, XRCC1, DNA ligase IIIα, and PARP1 are re-expressed, restoring downstream processing and ligation of BER/SSB intermediates. The resulting increase in repair capacity is accompanied by greater resistance to ROS-generating stress and improved cell survival. Thus, monocyte-to-macrophage differentiation selectively restores capacity to complete BER/SSB repair rather than uniformly increasing all components of the BER pathway. Created in BioRender. Jo, S. (2026) https://BioRender.com/m4oykk4.

Circulating human monocytes have limited capacity to complete BER and SSB repair. Enzymes involved in the early stages of BER, including OGG1, N-methylpurine DNA glycosylase (MPG), apurinic/apyrimidinic endonuclease 1 (APE1), and DNA polymerase β, remain detectable. Damaged bases can therefore be recognized, excised, and processed through the initial stages of BER. In contrast, X-ray repair cross-complementing protein 1 (XRCC1), DNA ligase IIIα, and poly(ADP-ribose) polymerase 1 (PARP1) are expressed at very low or undetectable levels [17]. BER initiation is therefore largely preserved, whereas downstream coordination and repair completion are impaired. Because base removal and incision generate strand-break intermediates that must subsequently be processed and sealed, inefficient completion can leave persistent SSB intermediates and increase monocyte sensitivity to oxidative stress. Consistent with this repair defect, circulating monocytes are highly susceptible to ROS-induced apoptosis, whereas macrophages and dendritic cells differentiated from them are comparatively resistant (Figure 2A) [17].

This unusually low repair capacity may therefore contribute to homeostatic regulation of the circulating monocyte pool by favoring the elimination of heavily damaged cells rather than their persistence within the myeloid compartment. Although this interpretation remains hypothetical, the similar downregulation of repair factors and increased damage sensitivity observed in mouse monocytes suggests that reduced repair capacity is a reproducible feature of the monocyte state rather than an incidental defect [18].

This repair defect is substantially reversed during differentiation into macrophages. XRCC1, DNA ligase IIIα, and PARP1 are re-expressed, accompanied by recovery of DNA ligation, PAR formation following oxidative stress, and more efficient resolution of BER/SSB intermediates [17]. Differentiation therefore restores the downstream steps required for BER/SSB completion rather than uniformly increasing all components of the pathway. The resulting increase in repair capacity is accompanied by greater resistance to ROS-generating stress (Figure 2B).

The re-expression of DNA repair factors is coupled to the macrophage differentiation program. EP300, HDAC1, and switch/sucrose non-fermenting (SWI/SNF) chromatin-remodeling complexes regulate expression of repair factors including PARP1, XRCC1, and breast cancer type 1 susceptibility protein (BRCA1) during macrophage differentiation. In macrophages, the balance between EP300- and HDAC1-dependent histone acetylation regulates recruitment of BRG1-containing SWI/SNF complexes to DNA repair gene promoters, linking differentiation-associated chromatin remodeling to restoration of repair factor expression [36].

A similar differentiation-dependent restoration extends to DNA-PK-dependent DSB repair. Circulating monocytes retain Ku70, Ku80, XRCC4, and DNA ligase IV but express very little DNA-PK catalytic subunit (DNA-PKcs), resulting in minimal DNA-PK activity and inefficient DSB repair following ionizing radiation. DNA-PKcs expression and DNA-PK activity increase during macrophage differentiation, together with increased resistance to ionizing radiation [17]. A comparable differentiation-dependent phenotype has also been observed in the mouse myeloid lineage, in which XRCC1, DNA ligase III, PARP1, and DNA-PKcs are reduced in monocytes and re-expressed after macrophage differentiation [18]. Together, these findings support differentiation-associated restoration of BER/SSB and DNA-PK-dependent DSB repair in the systems examined. However, whether comparable remodeling occurs in tissue-resident macrophages maintained through distinct developmental trajectories remains insufficiently defined.

The functional consequences of this differentiation-dependent shift in repair capacity may extend to ischemic tissues, where infiltrating monocytes and macrophages encounter intense oxidative stress during reperfusion. The differentiation studies establish an intrinsic defect in BER/SSB repair completion and sensitivity to oxidative stress [17,18], but do not show that this defect causes organ injury after ischemia. In experimental myocardial infarction, sequentially recruited monocyte subsets contribute to debris clearance and subsequent tissue repair [37]. By inference, excessive loss of reparative monocytes or macrophages could impair recovery, whereas prolonged survival of inflammatory cells could sustain injury; the net effect should depend on cell state and injury phase. A distinct mechanism operates in experimental hepatic ischemia–reperfusion, where oxidative DNA damage in hepatocytes promotes macrophage cyclic GMP–AMP synthase (cGAS)–stimulator of interferon genes (STING) signaling [38]. This parenchymal-to-myeloid communication should not be equated with defective repair within macrophages themselves. Determining whether restoration of BER/SSB repair protects organs will require lineage-resolved measurements of DNA lesions, repair kinetics, cell survival, and tissue recovery.

### 3.2. NER Attenuation During Differentiation

NER provides a clear contrast to the differentiation-associated restoration of BER/SSB and DNA-PK-dependent DSB repair [39]. During differentiation of human monocytes and monocytic cell lines toward macrophage states, global excision repair is substantially reduced in the differentiation models examined [19,35]. Macrophage differentiation therefore does not produce a common directional change across DNA repair pathways.

The extent of NER attenuation also depends on the lesion and genomic region examined. Repair of cisplatin-induced intrastrand crosslinks is strongly attenuated following macrophage-like differentiation, whereas removal of ultraviolet (UV)-induced 6-4 photoproducts remains comparatively proficient [35]. Cyclobutane pyrimidine dimers (CPDs) show a further distinction: their removal is inefficient when measured across the genome as a whole, whereas substantially greater repair is retained within transcriptionally active genes [19]. Reduced global NER therefore does not imply an equivalent loss of excision repair for every NER substrate or throughout all genomic regions.

Global NER attenuation is not explained simply by loss of canonical NER proteins. Biochemical complementation experiments identified the ubiquitin-activating enzyme E1 (E1/UBA1) as capable of restoring excision activity in repair-deficient macrophage extracts. Total E1 abundance changes little during differentiation, whereas its phosphorylation is reduced [19]. Transcription factor IIH (TFIIH) was proposed as a potential downstream target of this regulatory mechanism, but the relevant ubiquitination event and molecular sequence were not established. The E1/UBA1-TFIIH connection should therefore be regarded as a proposed mechanism rather than a defined differentiation-dependent pathway.

Much of the mechanistic evidence for this NER phenotype derives from ex vivo monocyte differentiation and monocytic leukemia-cell differentiation models studied before current genome-wide repair-mapping approaches became available [19,35]. These studies support selective attenuation of global NER in the systems examined, but evidence remains insufficient to determine how broadly this phenotype applies across primary tissue-resident macrophage populations.

### 3.3. Transcription Domain-Associated Repair During Macrophage Differentiation

The preservation of repair within transcriptionally active regions cannot be explained by canonical transcription-coupled NER (TC-NER) alone. Canonical TC-NER is initiated when elongating RNA polymerase II encounters a transcription-blocking lesion on the transcribed strand. Cockayne syndrome protein B (CSB), Cockayne syndrome protein A (CSA), and UV-stimulated scaffold protein A (UVSSA) subsequently coordinate recruitment of downstream NER machinery, including TFIIH, to the stalled transcription complex [40,41]. TC-NER is intrinsically strand specific because it is triggered when elongating RNA polymerase II encounters a lesion on the transcribed strand [42,43].

Following macrophage-like differentiation, CPDs are efficiently repaired on the transcribed strand of active genes, consistent with functional TC-NER. However, the non-transcribed strand of the same genes is also repaired more efficiently than the genome as a whole. This broader pattern, termed transcription domain-associated repair, therefore extends beyond canonical strand-specific TC-NER. These state-dependent changes in transcription and metabolism also influence the types and levels of DNA damage encountered by macrophages [44,45]. 

Macrophage differentiation thus alters not only the overall extent of NER but also its distribution across genomic regions. One possible explanation is that transcriptionally active chromatin remains more accessible to NER factors when global repair activity is reduced. However, this remains a model rather than an established macrophage mechanism. The original studies preceded current measurements of chromatin accessibility, three-dimensional genome organization, and genome-wide excision repair, and the molecular basis of transcription domain-associated repair therefore remains unresolved [19,46].

The preferential preservation of repair within transcriptionally active regions may be particularly relevant to the extensive inducible transcription described in Section 2.2. However, existing differentiation studies did not determine whether acute inflammatory activation dynamically redirects repair toward newly induced macrophage genes [19,35]. It therefore remains unclear whether preferential repair in transcriptionally active domains is a relatively stable property of active chromatin or is redistributed as macrophages alter their transcriptional state.

Together, these studies show that DNA repair capacity is altered in a pathway-specific manner during macrophage differentiation rather than uniformly increased or decreased. BER/SSB repair completion and DNA-PK-dependent DSB repair are restored, whereas global NER can be attenuated and excision repair is preferentially maintained within transcriptionally active genomic regions.

**Table 1:** Selective changes in DNA repair capacity during macrophage differentiation.

Repair Process		Macrophage Context	Repair Capacity	Mechanistic Basis	Biological Implication
BER/SSB repair		Monocyte → macrophage differentiation	Repair completion increased	Early BER enzymes retained; PARP1, XRCC1, and LigIIIα restored, enabling repair completion and ligation [17,18]	Greater resistance to oxidative stress
DNA-PK-dependent DSB repair			Increased	Restoration of DNA-PKcs and DNA-PK activity improves DSB rejoining [17,18]	Greater resistance to DSB/ionizing-radiation stress
NER	Global NER	Macrophage-like differentiation	Decreased	Decreased E1/UBA1 phosphorylation; involvement of TFIIH proposed [19,35]	No uniform increase in NER after differentiation
	TC-NER	Transcribed strand of active genes	Preserved	RNAPII stalling → CSB-CSA/UVSSA → TFIIH [19,35,40,41]	Strand-specific repair retained despite reduced global NER
Transcription domain-associated repair		Both strands of transcriptionally active domains	Preferentially retained	Active-chromatin accessibility is proposed; molecular basis remains unresolved [19,35,46]	Preferential repair of transcriptionally active genomic regions

## 4. DNA Repair and DDR Signaling in Macrophage Genome Maintenance

The pathway-specific differences described above become functionally important during macrophage activation and in disease-associated states. Depending on the predominant lesion or cellular context, distinct DNA repair and DDR pathways support macrophage survival, proliferation, inflammatory control, or senescence.

### 4.1. DNA Damage Responses in Disease-Associated Macrophages

**Figure 3:**
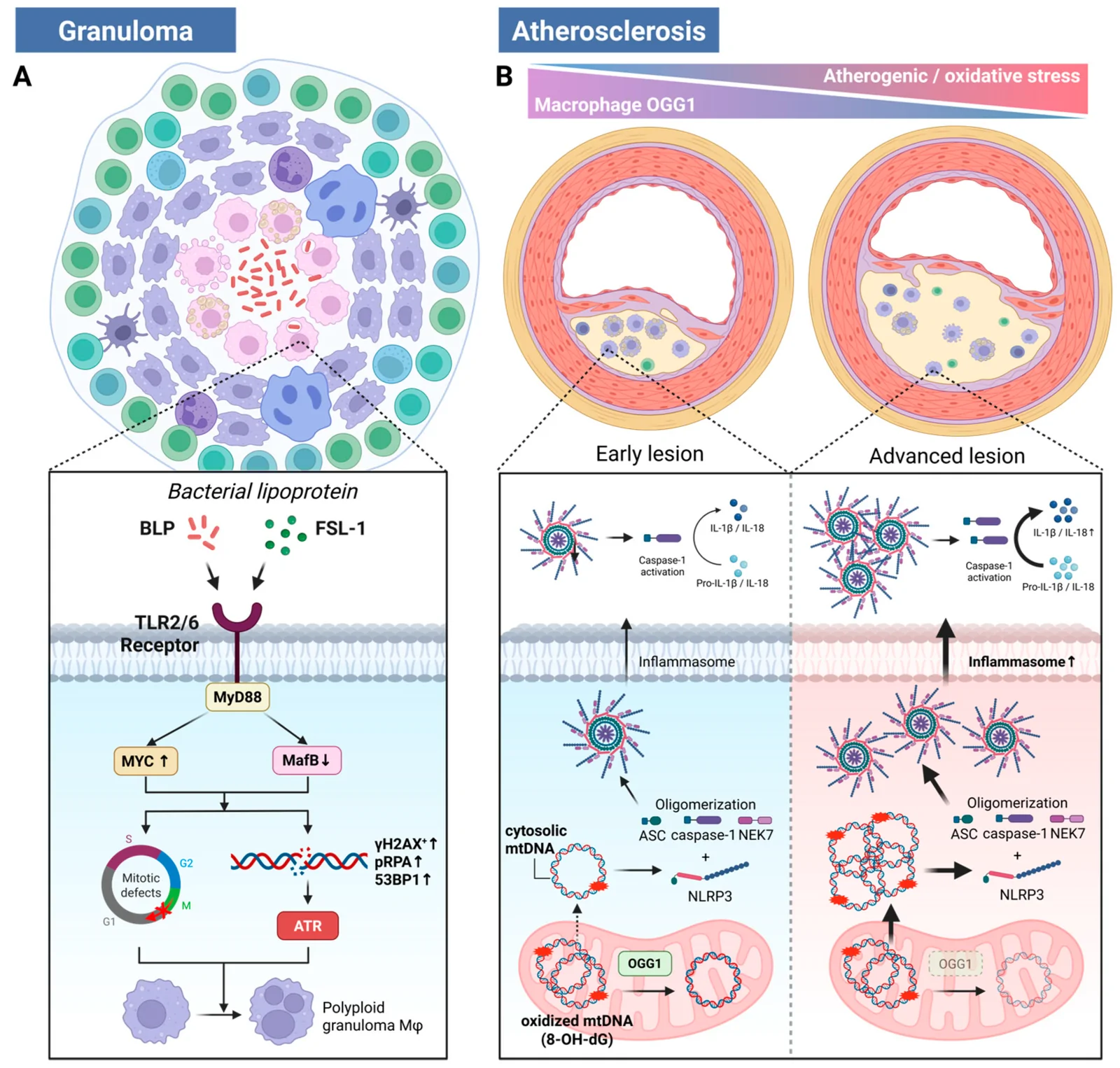
**DNA damage responses and repair in macrophages during granuloma formation and atherosclerosis.** (**A**) In granuloma-associated macrophages, persistent bacterial lipoprotein signaling through TLR2/6–myeloid differentiation primary response 88 (MyD88) promotes MYC-dependent cell-cycle progression together with reduced MafB expression. Repeated DNA replication is accompanied by slowed replication-fork progression, mitotic defects, and increased γH2AX, phosphorylated RPA (pRPA), and 53BP1. ATR-dependent DNA damage signaling limits chromosome-segregation errors during polyploid macrophage differentiation. (**B**) In atherosclerotic macrophages, increasing atherogenic and oxidative stress is associated with reduced OGG1 expression and accumulation of oxidized mitochondrial DNA (mtDNA). OGG1-dependent repair limits oxidized mtDNA accumulation, whereas OGG1 deficiency is associated with increased oxidized and cytosolic mtDNA, enhanced NLRP3 inflammasome activation, caspase-1 activation, and increased IL-1β and IL-18. Together, these models illustrate how distinct forms of DNA damage and replication stress engage different DNA damage signaling and repair mechanisms in disease-associated macrophages. Created in BioRender. Jo, S. (2026) https://BioRender.com/zv5gfku.

Granuloma-associated and atherosclerotic macrophages illustrate distinct forms of replication stress and oxidative DNA damage, respectively, and the cellular pathways that limit their consequences (Figure 3). In granulomas, persistent TLR2 signaling drives a specialized macrophage differentiation program involving MYC-dependent cell-cycle progression and repeated DNA replication. Under these conditions, replication-fork progression is slowed, accompanied by increased pRPA, γH2AX, and 53BP1 accumulation, providing evidence of replication stress [24]. Continued TLR2 signaling also promotes the formation of polyploid granuloma-associated macrophages through aberrant cell divisions and mitotic defects rather than cell fusion. ATR-dependent DNA damage signaling limits chromosome missegregation as additional genome copies accumulate [24]. Thus, ATR signaling restricts replication-associated chromosome instability during the development of polyploid granuloma macrophages (Figure 3A).

Atherosclerotic macrophages are exposed to lipid-associated oxidative stress. Intracellular free-cholesterol accumulation in primary macrophages increases mtROS, γH2AX, PARP1-dependent PARylation, and NAD^+^ consumption [47]. OGG1 removes oxidized guanine lesions and contributes to repair of oxidized mitochondrial DNA (mtDNA). In Ldlr-deficient mice fed a Western diet, Ogg1 expression decreases in lesion macrophages as atherosclerosis progresses, while OGG1 deficiency increases atherosclerotic plaque size and lipid accumulation [25]. Atherosclerotic lesions and macrophages from OGG1-deficient mice contain higher levels of oxidized mtDNA, and OGG1-deficient macrophages accumulate more cytosolic mtDNA and show increased caspase-1 activation and interleukin-1β (IL-1β) secretion. Circulating IL-1β and interleukin-18 (IL-18) are also increased in OGG1-deficient mice [25].

The inflammatory effects of OGG1 deficiency depend substantially on NLRP3. Transplantation of Ogg1-deficient bone marrow into Ldlr-deficient mice increases atherosclerotic lesion formation, whereas combined loss of OGG1 and NLRP3 reduces the increase in plaque size caused by OGG1 deficiency [25]. OGG1-dependent repair therefore limits oxidized mtDNA accumulation and its downstream activation of the NLRP3 inflammasome (Figure 3B).

Together, these models illustrate the context dependence of macrophage genome maintenance: ATR signaling primarily limits replication-associated chromosome instability, whereas OGG1 prevents oxidized mtDNA from amplifying inflammasome activation.

### 4.2. DNA Repair and Damage Signaling Support Macrophage Survival and Expansion

Beyond the lesion-specific responses observed in disease models, DNA repair and DDR signaling determine whether activated macrophages survive, proliferate, and contribute to tissue resolution. Pro-inflammatory stimulation induces DNA polymerase μ (Polμ) in a ROS-dependent manner. Loss of Polμ impairs repair of inflammation-associated DSBs, increases macrophage apoptosis, and reduces macrophage persistence at inflammatory sites and macrophage-dependent tissue regeneration [20]. In this setting, Polμ-dependent DSB repair supports macrophage survival under the same inflammatory conditions that generate DNA damage.

Macrophages undergoing efferocytosis engage a different protective response. A DNMT3A-dependent pathway increases PARP1 chromatin association and PARylation and promotes the nuclear accumulation of MTH1/NUDT1, reduces oxidized DNA burden, and supports macrophage proliferation and tissue resolution [48,49]. This response links clearance of apoptotic cells with control of oxidized DNA while macrophages continue tissue-repair functions.

DNA damage signaling is also important when macrophages expand. Proliferative stimulation and IFN-γ/LPS treatment increase NBS1 abundance, whereas macrophages carrying a hypomorphic Nbs1 allele accumulate more DNA damage, proliferate less efficiently, differentiate more slowly, and show increased senescence and pro-inflammatory cytokine expression [50]. Nbs1 deficiency also markedly reduces macrophage proliferation during sterile peritoneal inflammation in vivo [50]. Although these observations do not identify a specific replication lesion repaired by NBS1, they show that NBS1-dependent DNA damage signaling is required for efficient macrophage proliferation under inflammatory conditions.

### 4.3. DNA-PK–STAT6 Signaling in Genome Maintenance and Senescence

Persistent DNA damage can promote macrophage senescence rather than immediate cell death. Interleukin-4 (IL-4) signaling counteracts this response through STAT6-dependent expression of DNA repair genes. STAT6 promotes the expression of genes involved in homologous recombination and the Fanconi anemia pathway, including BRCA1, RAD51, FANCD2, FANCI, and UBE2T. Loss of STAT6 reduces the expression of these DNA repair genes, increases DNA damage and DNA fragments in the cytoplasm, and is accompanied by greater macrophage senescence and tissue inflammation. Conversely, IL-4 treatment reduces macrophage senescence, linking type 2 cytokine signaling to the preservation of genome integrity [21].

A subsequent study identified DNA-PK as a direct regulator of this STAT6-dependent repair response. Following DNA damage, DNA-PK phosphorylates murine STAT6 at Ser807, corresponding to Ser817 in human STAT6. This phosphorylation reduces RNF128-dependent ubiquitination and degradation of STAT6, allowing activated STAT6 to persist. Phosphorylated STAT6 also cooperates with the macrophage lineage transcription factor PU.1 at DNA repair gene loci, increasing expression of BRCA1 and UBE2T and improving DNA repair [51]. DNA-PK-dependent phosphorylation does not replace canonical IL-4-dependent STAT6 activation; instead, it stabilizes activated STAT6 and enhances STAT6-dependent transcription of DNA repair genes.

Disruption of this pathway increases γH2AX and is accompanied by reduced proliferation and multiple features of macrophage senescence [51]. The IL-4-STAT6 and DNA-PK-STAT6 pathways therefore connect cytokine signaling and the DNA damage response to DNA repair gene expression. When this connection is impaired, DNA damage persists and macrophages are more likely to develop a senescent, pro-inflammatory phenotype.

These findings also connect polarization-associated signals to genome maintenance. IFN-γ/LPS activation generates ROS-associated DNA damage and engages repair-dependent survival mechanisms (Section 2.1 and Section 4.2), whereas IL-4–STAT6 signaling promotes DNA repair gene expression and limits senescence [20,21,29,51]. This contrast does not establish that all M2-like macrophages repair DNA more efficiently than M1-like macrophages. Such comparisons require the same lesion, macrophage source, cell-cycle distribution, and repair assay under defined activation conditions.

DNA repair and DNA damage signaling can therefore support macrophage survival and proliferation while limiting senescence under distinct genotoxic conditions. When damaged DNA persists or accumulates outside its normal nuclear or mitochondrial context, it can also alter inflammatory signaling and communication with other immune cells, as discussed in the following section.

## 5. DNA Damage-Driven Inflammatory and Intercellular Signaling

DNA damage can alter macrophage inflammatory and intercellular signaling through three related routes. First, damaged or mislocalized self-DNA can activate cytosolic sensing pathways. Second, repair deficiency can alter extracellular-vesicle production and cargo. Third, persistent nuclear damage can redirect self-derived material into the MHC-II presentation pathway. Together, these mechanisms connect intracellular genome maintenance to neighboring cells and the adaptive immune system.

### 5.1. Self-DNA Sensing and Inflammatory Signaling

**Figure 4:**
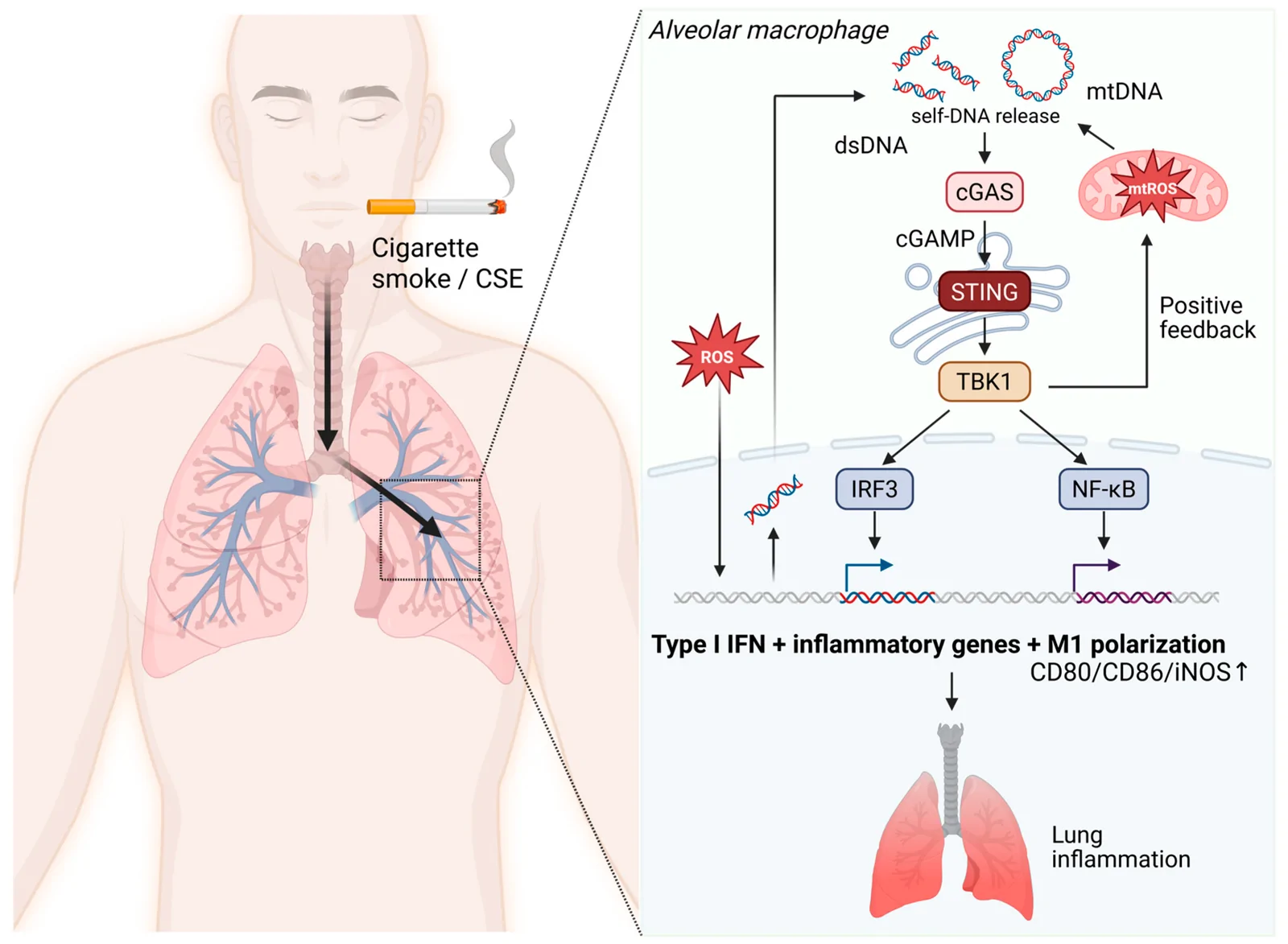
**Cigarette-smoke-induced self-DNA release activates macrophage cGAS/STING/TBK1 signaling.** Cigarette smoke extract (CSE) increases mitochondrial reactive oxygen species (mtROS), promoting the overproduction and release of double-stranded DNA (dsDNA) and mitochondrial DNA (mtDNA). Self-DNA activates cGAS/STING/TBK1 signaling and downstream IRF3/NF-κB pathways, increasing CD80, CD86, iNOS, type I interferons, and pro-inflammatory cytokines. Released dsDNA further increases mtROS production and mtDNA release, reinforcing the inflammatory response. Genetic or pharmacological disruption of cGAS/STING/TBK1 signaling, inhibition of dsDNA release, or reduction in mtROS attenuates these responses. Created in BioRender. Jo, S. (2026) https://BioRender.com/kcd4n2g.

Cigarette smoke extract (CSE) increases mtROS in macrophages and promotes the overproduction and release of double-stranded DNA (dsDNA) and mitochondrial DNA (mtDNA) [52]. Activation of macrophage-intrinsic cyclic GMP–AMP synthase (cGAS)/stimulator of interferon genes (STING)/TANK-binding kinase 1 (TBK1) signaling and downstream interferon regulatory factor 3 (IRF3)/nuclear factor kappa B (NF-κB) pathways increases CD80, CD86, and inducible nitric oxide synthase (iNOS) expression together with type I interferons and pro-inflammatory cytokines. Genetic loss of macrophage cGAS or TBK1, STING deficiency, inhibition of dsDNA release, or reduction in mtROS attenuates these responses. Released dsDNA also promotes further mtROS production and mtDNA release, reinforcing the inflammatory response under CSE exposure (Figure 4) [52].

Cytoplasmic DNA sensing is also increased when DNA damage responses are impaired. ATM-deficient macrophages contain more cytoplasmic DNA and show increased type I interferon signaling. Irradiation similarly increases cytoplasmic DNA in wild-type macrophages, whereas loss of stimulator of interferon genes (STING) reduces the interferon response without preventing DNA accumulation [53]. These findings show that self-DNA generated after genotoxic stress can activate STING-dependent type I interferon signaling in macrophages [54].

Together, the CSE-exposure and ATM-deficiency models show that self-DNA can function as an inflammatory signal when it is released or accumulates outside the nucleus or mitochondria. In these settings, cGAS/STING/TBK1 or STING-dependent signaling converts self-DNA accumulation into macrophage inflammatory responses.

### 5.2. Extracellular-Vesicle-Mediated Communication

Persistent DNA damage can also alter macrophage communication with neighboring cells. In macrophages with ERCC1-XPF repair deficiency, accumulated DNA damage is accompanied by Golgi dispersal, endoplasmic-reticulum dilation, increased autophagy, and enhanced extracellular-vesicle (EV) production. These EVs are taken up by neighboring cells, where they increase glucose uptake, oxygen consumption, and inflammatory signaling [22]. Thus, DNA damage within macrophages can alter the metabolism and inflammatory activity of nearby cells through EV-mediated communication.

### 5.3. Repair Failure and Self-Antigen Presentation

Persistent DNA damage can also alter the self-derived peptides that macrophages present to CD4^+^ T cells. In macrophages with ERCC1-XPF repair deficiency, nuclear and ribosomal proteins accumulate in autophagosomal and lysosomal compartments. Autophagy delivers this material to the major histocompatibility complex class II (MHC-II) processing pathway, producing an MHC-II peptide repertoire enriched in nuclear and ribosomal self-peptides [23,55,56].

This change in antigen presentation has measurable immune consequences. ERCC1-XPF-deficient macrophages stimulate stronger T-cell responses, while mice with myeloid ERCC1-XPF repair deficiency develop polyclonal T-cell activation, antinuclear autoantibodies, immune-complex deposition, and inflammatory pathology. Blocking autophagy reduces nuclear-antigen delivery and suppresses several autoimmune features, showing that autophagic processing of nuclear material is an important step connecting macrophage DNA damage to autoreactivity [23].

Repair failure can therefore alter not only inflammatory signaling but also the antigenic information presented by macrophages. Persistent nuclear damage increases the entry of self-derived nuclear material into the MHC-II pathway, linking macrophage DNA damage to autoreactive adaptive immune responses.

Taken together, these findings distinguish inflammatory sensing of mislocalized DNA from repair-deficiency-associated changes in secretion and self-antigen presentation. Cytosolic DNA sensing changes macrophage inflammatory output, extracellular vesicles transmit metabolic and inflammatory effects to neighboring cells, and altered MHC-II presentation can engage autoreactive T cells. Thus, the immune consequences of persistent or mislocalized DNA damage depend on how damaged cellular material is compartmentalized, released, or processed. These mechanisms were established in different experimental settings and should not be assumed to occur together in every damaged macrophage. They nevertheless provide complementary routes by which failed genome maintenance can extend beyond the affected cell (Table 2).

## 6. Circadian Regulation of Macrophage Activity and Implications for DNA Repair

The preceding sections describe genome maintenance as a function of macrophage identity and activation state. A further question is whether DNA damage formation, repair, and downstream inflammatory signaling also vary with circadian time. Macrophage metabolism, trafficking, phagocytosis, and inflammatory activity all exhibit time-of-day variation [26,57,58,59]. These findings establish broad circadian regulation of macrophage and myeloid-cell function but do not demonstrate rhythmic DNA repair in macrophages [60,61]. The following sections therefore distinguish established clock-dependent macrophage functions, repair mechanisms demonstrated in other systems, and testable links between them.

### 6.1. Macrophage Clock and Immunometabolism

Inflammatory macrophage activation is accompanied by increased glucose utilization and metabolic reprogramming. BMAL1 constrains glucose uptake and glycolytic and Krebs-cycle flux; loss of Bmal1 increases glycolysis and succinate accumulation and enhances nuclear PKM2–STAT3 signaling, leading to increased pro-IL-1β expression [62].

**Figure 5:**
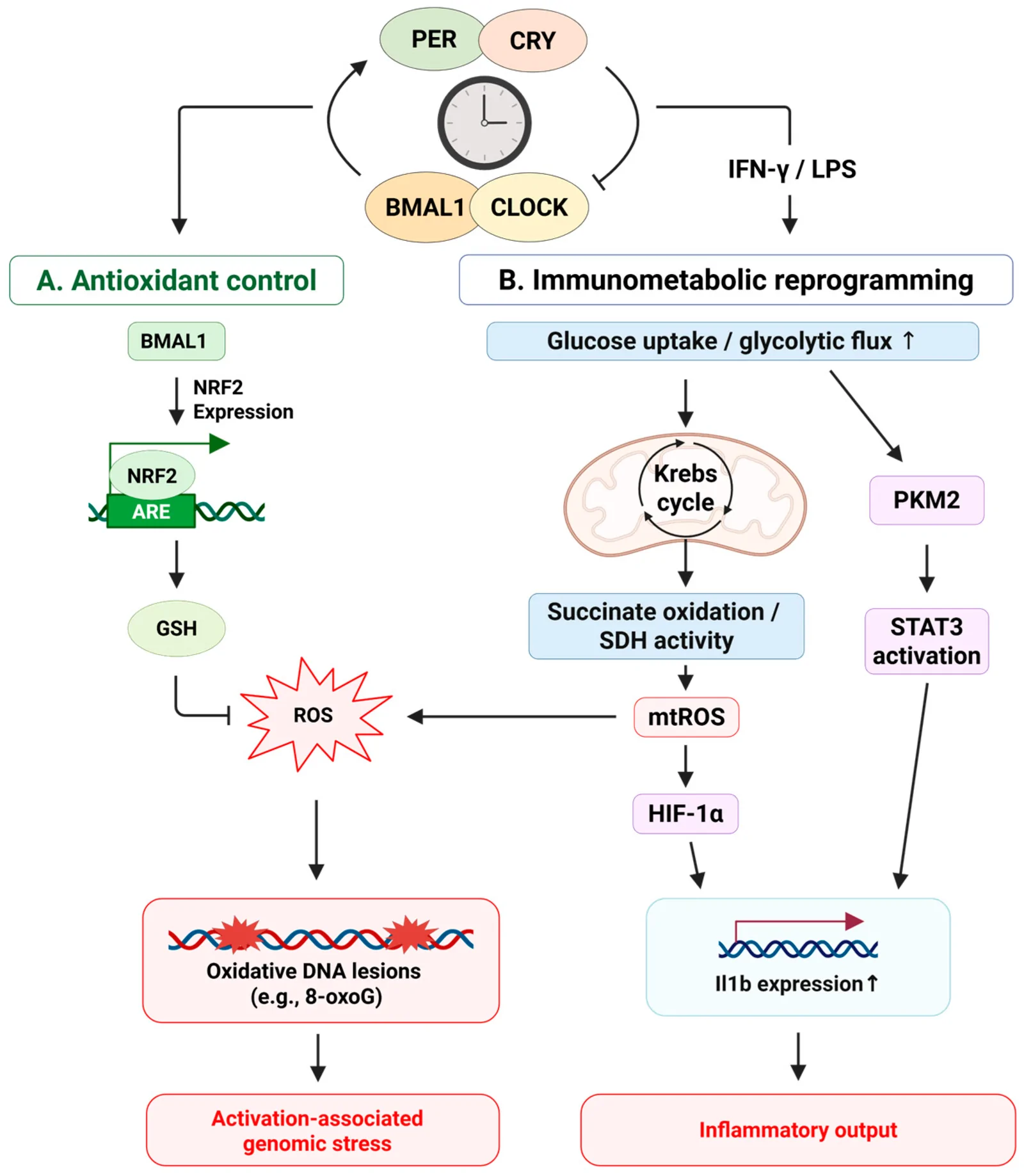
**Circadian regulation of antioxidant defense and immunometabolic stress in activated macrophages.** The macrophage circadian clock coordinates redox control with inflammatory metabolic reprogramming. BMAL1 promotes NRF2-dependent antioxidant responses, including glutathione (GSH) production, thereby limiting intracellular ROS accumulation. When ROS exceeds antioxidant capacity, oxidative DNA lesions, including 8-oxoguanine (8-oxoG), can accumulate during macrophage activation. In parallel, IFN-γ/LPS stimulation increases glucose utilization and glycolytic flux and promotes mitochondrial metabolic remodeling. Enhanced succinate oxidation through succinate dehydrogenase (SDH) increases mitochondrial ROS (mtROS), which contributes to HIF-1α-dependent inflammatory gene expression, while PKM2-dependent STAT3 activation provides an additional route supporting Il1b expression. The redox and immunometabolic pathways are supported by macrophage studies; their proposed circadian linkage to oxidative DNA lesion burden remains to be tested directly. The figure does not establish rhythmic DNA repair activity in macrophages. Created in BioRender. Jo, S. (2026) https://BioRender.com/rzmqcsw.

BMAL1 also regulates mitochondrial redox signaling. During IFN-γ/LPS-induced activation, loss of BMAL1 increases succinate accumulation and succinate dehydrogenase-dependent mitochondrial ROS, which stabilizes hypoxia-inducible factor 1α (HIF-1α) and promotes glycolytic and inflammatory reprogramming [27]. In parallel, BMAL1 drives circadian nuclear factor erythroid 2-related factor 2 (NRF2)-dependent antioxidant activity during LPS stimulation. Higher NRF2 activity is associated with lower ROS accumulation and reduced HIF-1α-dependent IL-1β production [63]. BMAL1 therefore connects metabolic substrate utilization, mitochondrial ROS production and antioxidant defense in activated macrophages (Figure 5).

Mitochondrial inflammatory signaling also varies with circadian phase. Peritoneal macrophages collected at CT12 (circadian time 12), the onset of the subjective active phase, show higher mitochondrial membrane potential and stronger NLRP3 inflammasome activation than those collected at CT0. This is accompanied by greater CASP1 and GSDMD activation, IL-1β release and pyroptotic cell death [64].

Together, these studies show that macrophage metabolism, redox activity, mitochondrial signaling and inflammatory responses vary with circadian time [65,66,67]. Because these processes can promote DNA damage, lesion formation may likewise vary across the day. Whether DNA repair capacity shows comparable circadian regulation in macrophages remains unknown.

### 6.2. Circadian DNA Repair in Other Systems

Direct evidence for circadian variation in DNA repair capacity comes primarily from non-macrophage systems. In mouse brain and liver, NER activity varies with circadian time in parallel with xeroderma pigmentosum group A protein (XPA) abundance [68,69]. In liver, rhythmic XPA abundance results from circadian transcription combined with HERC2-dependent protein turnover, linking a rate-limiting NER factor directly to circadian changes in repair capacity [69,70].

Diurnal variation has also been reported for oxidative DNA lesion repair. In human peripheral lymphocytes, OGG1 expression and activity vary with time of day together with changes in 8-oxoguanine accumulation and repair [71]. Beyond oxidative lesion repair, clock proteins may also participate directly in the processing of specific DNA lesions. For example, PER2, a core clock protein, is recruited to transcription-associated double-strand breaks and promotes their anchoring to the nuclear envelope through SUN1 and NUP153, which facilitates RAD51-mediated homologous recombination [72].

These studies identify circadian regulation of repair factor abundance and direct repair functions of clock proteins in non-macrophage systems. However, these observations do not establish circadian oscillation of the corresponding repair pathways, and their phase and amplitude in macrophages remain to be determined directly.

### 6.3. Potential Circadian Coordination of Genome Maintenance

Three pathway-specific links provide a framework for testing circadian regulation of macrophage genome maintenance: oxidative DNA lesion processing, transcription-associated stress, and replication stress.

The most direct potential link involves oxidative DNA lesion processing. BMAL1-dependent metabolic and NRF2 programs influence ROS production and antioxidant defense [27,62,63]. Complementary macrophage studies show that differentiation restores BER/SSB repair completion and that OGG1-dependent repair limits oxidized mitochondrial DNA [17,18,25]. These observations motivate testing whether nuclear and mitochondrial lesion burdens vary with the relative timing of ROS production, base excision, and strand-break sealing. A rhythm in ROS or OGG1 abundance alone would not establish rhythmic completion of repair. Conversely, phase-dependent lesion persistence could arise from changing lesion formation even if repair capacity remains constant.

A second potential link involves transcription-associated stress. TOP1-dependent relaxation supports inducible transcription [15,33], but relaxation of torsional stress is not itself lesion excision. When transcription is blocked by a suitable bulky lesion, TC-NER can protect the transcribed strand, while differentiation models show preferential repair across active domains [19,35]. A temporal change in inflammatory transcription could therefore alter where repair is needed without increasing global NER. Rhythmic XPA-dependent NER in brain or liver [68,69,70] provides a rationale for testing this possibility, not evidence that macrophages share the same rhythm. Most oxidative base lesions instead require BER, so rhythmic redox activity should not be treated as a direct readout of NER.

A third potential link concerns replication stress in proliferating macrophages. The ATR response in granuloma-associated macrophages [24] provides a basis for asking whether circadian phase affects fork stress or checkpoint engagement, but does not establish a circadian ATR rhythm. Likewise, repair mechanisms requiring a sister chromatid cannot be extrapolated from cycling cells to quiescent macrophages. Overall, circadian coordination is a testable, pathway-specific hypothesis: repair may coincide with, anticipate, or follow periods of increased stress, and its phase relationship must be measured rather than assumed. Any mismatch could affect lesion persistence and the inflammatory communication described in Section 5, but this downstream temporal relationship also remains untested.

## 7. Experimental Gaps and Future Directions

The preceding sections identify state-dependent sources of DNA damage, replication stress, and transcription-associated stress, selective restoration of BER/SSB and DNA-PK-dependent repair during differentiation, and preferential retention of excision repair in active genomic regions. They also show that persistent damage or mislocalized DNA can alter inflammatory communication. Circadian regulation adds evidence for temporal changes in macrophage metabolism and redox activity, but its coupling to pathway-specific lesion removal remains unresolved. The central experimental task is therefore to determine how macrophage identity, activation state, and time influence damage formation and repair in the same defined cell population.

First, DNA repair capacity should be compared across macrophages of distinct developmental origins and tissue environments. Tissue-resident macrophages acquire distinct transcriptional and chromatin profiles in response to their local environments [4,6,7], but corresponding differences in DNA damage formation and repair have rarely been measured directly. Comparative analysis should include Kupffer cells, microglia, splenic and connective-tissue macrophages, alongside monocyte-derived macrophages. Such comparisons should distinguish developmental origin and tissue identity from experimentally induced M1/M2-like activation, using defined stimuli and matched lesion-specific assays.

Second, lesion formation must be distinguished experimentally from repair kinetics and persistent DDR signaling. Increased γH2AX, altered repair factor abundance, or changes in inflammatory output cannot by themselves determine whether macrophages generate more lesions, repair them more slowly, or sustain DDR signaling after lesion removal. Lesion-specific measurements should therefore be combined with pathway-specific repair assays and functional readouts in the same macrophage population. DNA damage and persistent DNA damage response (DDR) signaling should also be distinguished from stable genomic alterations such as mutations or chromosomal abnormalities.

Third, genome- and strand-resolved approaches are needed to define the genomic distribution of repair. The preferential repair of transcriptionally active genomic regions observed in macrophage differentiation models indicates that bulk measurements can obscure substantial differences in repair across the genome. Methods such as excision repair sequencing can map repair according to lesion type, genomic position, and transcriptional strand [73,74]. Combining these approaches with nascent transcription and chromatin accessibility measurements could determine whether macrophage activation changes where repair occurs and whether newly induced genes exhibit preferential repair.

Finally, genome maintenance should be examined longitudinally rather than at single endpoints. Lesion formation, DDR activation, repair, transcriptional recovery, and changes in macrophage function occur on different timescales. Time-resolved measurements are therefore needed to distinguish increased lesion formation from delayed repair or incomplete recovery. Extending these measurements across repeated inflammatory exposures could further determine whether macrophages fully recover between episodes of activation or progressively accumulate persistent DNA damage.

Testing circadian coordination will require lesion formation and removal to be measured at multiple phases over more than one circadian cycle. Initial lesion burden, viability, activation state, and cell-cycle composition must be accounted for. Clock perturbation should be combined with rescue and time-series analysis, because the phenotype resulting from Bmal1 deficiency may reflect nonrhythmic functions as well as clock disruption. Nuclear and mitochondrial oxidative DNA lesion measurements, BER/SSB completion assays, lesion- and strand-resolved NER measurements, and fork/checkpoint analyses in cycling cells can then test the pathway-specific relationships proposed in Section 6.3.

## 8. Conclusions

Genome maintenance during macrophage differentiation and inflammatory activation depends on the types of DNA lesions, replication stress, and transcription-associated challenges encountered in each cellular state, together with the repair and signaling pathways available to manage them. Differentiation models reveal selective restoration of BER/SSB and DNA-PK-dependent repair alongside attenuation of global NER and preferential repair of active genomic regions. During activation, DNA repair and DDR signaling support macrophage survival and proliferation while limiting senescence, while persistent or mislocalized DNA can alter inflammatory signaling, extracellular-vesicle communication, and self-antigen presentation. These findings should not be generalized to every tissue-resident population or M1/M2-like state. Circadian regulation of metabolism and redox activity suggests a temporal dimension, but circadian regulation of DNA repair capacity has not yet been established in macrophages. Defining this balance requires matched, lesion-specific and time-resolved measurements across clearly identified macrophage populations.

## Figures and Tables

**Table 2:** Functional and immunological consequences of DNA damage and altered genome maintenance in macrophages.

Pathway or Defect	Mechanism	Macrophage Outcome	Tissue/Immune Consequence
IL-4–STAT6 signaling	STAT6-dependent HR/FA DNA repair gene expression [21]	Reduced DNA damage and cytoplasmic nuclear DNA; reduced senescence	Reduced tissue inflammation and age-associated dysfunction
DNA-PK–STAT6 signaling	STAT6 Ser807 phosphorylation → reduced degradation; increased BRCA1/UBE2T expression [51]	Enhanced DNA repair; reduced γH2AX and senescence	Reduced inflammaging-associated phenotypes
CSE-induced self-DNA–cGAS/STING/TBK1 signaling	CSE → increased mtROS → dsDNA/mtDNA release → cGAS-STING-TBK1 → IRF3/NF-κB [52]	Increased CD80/CD86/iNOS, type I IFN, and pro-inflammatory cytokines	Increased cigarette-smoke-induced lung inflammation
Cytosolic DNA–STING signaling	Cytoplasmic DNA accumulation → STING activation [53]	Increased type I IFN signaling	Increased innate inflammatory output
ERCC1–XPF deficiency: EV axis	Persistent DNA damage → organelle remodeling/autophagy → increased EV release [22]	Altered EV production and cargo	Metabolic and inflammatory changes in neighboring cells
ERCC1–XPF deficiency: MHC-II axis	Nuclear material → autophagy → altered MHC-II immunopeptidome [23]	Increased nuclear/ribosomal self-peptide presentation	Increased CD4^+^ T-cell activation and autoreactivity

## Data Availability

No new data were created or analyzed in this study. Data sharing is not applicable to this article.

## Abbreviations

The following abbreviations are used in this manuscript:

8-OHdG	8-Hydroxy-2′-deoxyguanosine
8-oxoG	8-Oxoguanine
53BP1	p53-binding protein 1
AM	Alveolar macrophage
AMPK	AMP-activated protein kinase
ARDS	Acute respiratory distress syndrome
ATM	Ataxia-telangiectasia mutated
ATR	Ataxia-telangiectasia and Rad3-related
BER	Base excision repair
BMAL1	Brain and muscle ARNT-like 1
BRCA1	Breast cancer type 1 susceptibility protein
CD5L	CD5 antigen-like
cGAS	Cyclic GMP-AMP synthase
CIITA	Class II major histocompatibility complex transactivator
CLEC4F	C-type lectin domain family 4 member F
CPD	Cyclobutane pyrimidine dimer
CSE	Cigarette smoke extract
CSA	Cockayne syndrome protein A
CSB	Cockayne syndrome protein B
CT	Circadian time
CX3CR1	C-X3-C motif chemokine receptor 1
DDR	DNA damage response
DNA	Deoxyribonucleic acid
DNA-PK	DNA-dependent protein kinase
dsDNA	Double-stranded DNA
DNA-PKcs	DNA-dependent protein kinase catalytic subunit
DSB	DNA double-strand break
E1/UBA1	Ubiquitin-activating enzyme E1
EGR2	Early growth response 2
EpCAM	Epithelial cell adhesion molecule
ERCC1	Excision repair cross-complementation group 1
EV	Extracellular vesicle
FA	Fanconi anemia
GM-CSF	Granulocyte-macrophage colony-stimulating factor
HIF-1α	Hypoxia-inducible factor 1α
HR	Homologous recombination
HSE	Human skin equivalent
IFN-γ	Interferon gamma
IL-1β	Interleukin-1β
IL-4	Interleukin-4
IRF3	Interferon regulatory factor 3
IL-10	Interleukin-10
IL-18	Interleukin-18
LPS	Lipopolysaccharide
LYVE1	Lymphatic vessel endothelial hyaluronan receptor 1
MARCO	Macrophage receptor with collagenous structure
MHC-II	Major histocompatibility complex class II
MRN	MRE11–RAD50–NBS1
mtROS	Mitochondrial reactive oxygen species
mtDNA	Mitochondrial DNA
MyD88	Myeloid differentiation primary response 88
NAD^+^	Nicotinamide adenine dinucleotide, oxidized form
NAMPT	Nicotinamide phosphoribosyltransferase
NBS1	Nijmegen breakage syndrome protein 1
NER	Nucleotide excision repair
NF-κB	Nuclear factor kappa B
NHEJ	Non-homologous end joining
NLRP3	NOD-like receptor family pyrin domain containing 3
NRF2	Nuclear factor erythroid 2-related factor 2
OGG1	8-Oxoguanine DNA glycosylase
PARP	Poly(ADP-ribose) polymerase
PARP1	Poly(ADP-ribose) polymerase 1
PM	Particulate matter
Polμ	DNA polymerase μ
PPARγ	Peroxisome proliferator-activated receptor gamma
RNA	Ribonucleic acid
RNS	Reactive nitrogen species
ROS	Reactive oxygen species
RPA	Replication protein A
SiglecF	Sialic acid-binding immunoglobulin-like lectin F
SSB	DNA single-strand break
STAT6	Signal transducer and activator of transcription 6
TBK1	TANK-binding kinase 1
STING	Stimulator of interferon genes
SWI/SNF	Switch/sucrose non-fermenting chromatin-remodeling complex
TC-NER	Transcription-coupled nucleotide excision repair
TFIIH	Transcription factor IIH
TGF-β	Transforming growth factor beta
TIM4	T-cell immunoglobulin and mucin domain-containing protein 4
TLR	Toll-like receptor
TLR2	Toll-like receptor 2
TOP1	DNA topoisomerase I
UV	Ultraviolet
UVA	Ultraviolet A
UVSSA	UV-stimulated scaffold protein A
VSIG4	V-set and immunoglobulin domain-containing protein 4
XPA	Xeroderma pigmentosum group A protein
XPF	Xeroderma pigmentosum group F protein
XRCC1	X-ray repair cross-complementing protein 1
γH2AX	Phosphorylated histone H2AX
